# Factors associated with diabetic retinopathy: a cross-sectional study of 1477 patients across four cities in Anhui, China

**DOI:** 10.3389/fendo.2026.1922694

**Published:** 2026-09-22

**Authors:** Yanlei Wang, Liya Ai, Pan Yang, Ying Xing, Huaiyu Lin, Shumin Wang, Guilong Sun, Ying Zhang, Liping Xue, Tian Jiang, Qiu Zhang, Yi Zhang

**Affiliations:** 1 Department of Endocrinology, The First Affiliated Hospital of Anhui Medical University, Hefei, Anhui, China; 2 Department of Endocrinology, The Second Affiliated Hospital of Nanjing Medical University, Nanjing, Jiangsu, China

**Keywords:** associated factors, cross-sectional study, diabetic retinopathy, HbA1c, UACR

## Abstract

**Background:**

Diabetic retinopathy (DR) remains the leading cause of blindness among working-age adults. With the rising prevalence of diabetes, the burden of DR continues to escalate, substantially impairing patients’ quality of life. Identifying clinical factors associated with prevalent DR may help refine risk-based ophthalmic assessment. Using a large cross-sectional dataset, this study examined associations between routinely available clinical indicators and the presence of DR. Particular attention was given to heart rate (HR), an understudied and readily available vital sign, and to diabetes treatment histories.

**Methods:**

Leveraging data from the China DiaChronic Study, this cross-sectional analysis included data from 1,477 patients with diabetes. Categorical variables (sex, smoking and alcohol status, education) were analyzed by chi-square tests; continuous variables by independent-samples t-tests or Wilcoxon rank-sum tests. Binary logistic regression estimated factors associated with prevalent DR after adjustment for demographic, metabolic, diabetes-duration, and treatment-history variables; variance inflation factors (VIFs) were used to assess multicollinearity; ROC curves described cross-sectional discrimination; formal interaction terms assessed whether selected associations differed by HbA1c category; and Spearman correlation assessed relationships between HbA1c and other parameters.

**Results:**

Among 1,477 patients, 259 (17.5%) had DR. After additional adjustment for diabetes duration and medication histories, higher systolic blood pressure (SBP), heart rate (HR), HbA1c, blood urea nitrogen (BUN), urinary albumin-creatinine ratio (UACR), and longer diabetes duration were associated with prevalent DR (all *P* < 0.05), with VIFs ranging from 1.07 to 1.59. HbA1c showed the greatest cross-sectional discrimination (AUC 0.672, 95% CI 0.637-0.707), followed by UACR (AUC 0.655, 95% CI 0.617–0.692), although both AUCs were modest. Exploratory stratification by HbA1c <6.5% (*n* = 446, DR = 37) versus ≥6.5% (*n* = 1,031, DR = 222) showed different within-stratum patterns, but formal interaction tests for SBP, HR, BUN, and UACR were not statistically significant (all *P* for interaction >0.05). Spearman correlation showed FBG, HR, BUN, and UACR positively correlated with HbA1c (*r* = 0.094-0.641, *P* < 0.05), with UACR exhibiting the strongest association after FBG (*r* = 0.216, *P* < 0.001).

**Conclusion:**

Higher SBP, HR, HbA1c, BUN, UACR, and longer diabetes duration were associated with prevalent DR after multivariable adjustment. Among these, the association with HR is the most potentially novel and may reflect cardiac autonomic dysfunction rather than a direct causal effect. Although HbA1c and UACR showed only modest cross-sectional discrimination, the findings support integrated cardiometabolic, renal, and diabetes-history assessment to complement guideline-recommended retinal screening. Prospective validation is warranted.

## 1 Background

Diabetic retinopathy (DR), as one of the most critical microvascular complications of diabetes, represents the primary cause of visual impairment and blindness in working-age adults (1, 2). Its disease burden escalates alongside the rising prevalence of diabetes. Studies demonstrate a significant positive correlation between DR incidence and diabetes duration: 1.11% within 2.5 years, 9.04% at 2.5-5.0 years, and 28.14% beyond 5 years (3). In early stages, DR lacks typical clinical symptoms and requires specialized fundus examination to detect microaneurysms or dot-blot hemorrhages. Progressive stages manifest retinal edema, venous beading, and intraretinal microvascular abnormalities. Advanced disease triggers pathological neovascularization, culminating in vitreous hemorrhage and tractional retinal detachment, which ultimately cause irreversible blindness. This severely compromises life quality of patients and imposes substantial socioeconomic burden (4).

The pathogenesis of DR is multifactorial, centrally involving chronic hyperglycemia-induced inflammation, immune dysregulation, and metabolic disturbance (5). Key mechanisms include: Dysregulation of polyol pathways (6), Protein kinase C (PKC) activation, Advanced glycation end product (AGE) accumulation (1) and Upregulation of vascular endothelial growth factor (VEGF) (7, 8). These pathways collectively drive breakdown of the blood-retinal barrier, retinal ischemia/hypoxia, and neurovascular unit dysfunction. Emerging evidence on the gut-retina axis suggests that pro-inflammatory metabolites derived from gut microbial dysbiosis exacerbate DR pathological progression (9, 10). Advancing research continues to refine screening and diagnostic approaches for DR. Clinicians now utilize multimodal imaging modalities such as fundus photography, fluorescein angiography (FA), optical coherence tomography (OCT), and OCT angiography (OCTA) (11) to precisely stage DR severity. Concurrently, artificial intelligence (AI) tools including artificial neural networks (ANN) and transfer learning techniques have demonstrated emerging capabilities in distinguishing healthy retinas from DR pathology (12). Therapeutic interventions for DR are stratified by disease severity. Current strategies include laser photocoagulation, intravitreal anti-VEGF agents, corticosteroid implants, and vitrectomy. Nanoparticle-based drug delivery systems show promising potential for mitigating treatment-related adverse effects (13).

In a series of studies on DR, numerous scholars have focused on analyzing associated risk factors. According to existing cross-sectional and prospective cohort studies, in addition to well-established factors such as duration of diabetes and glycated hemoglobin(HbA1c)levels, clinical risk factors including hypertension (14), dyslipidemia (15), unhealthy lifestyle behaviors (16), socioeconomic status (17), and genetic predisposition (5) have been consistently linked to the onset and progression of DR. Moreover, long-term exposure to environmental pollutants such as particulate matter and nitrogen dioxide has also been associated with an increased risk of DR (18). Although factors related to DR have been extensively studied, the relative contribution of routinely available glycemic, hemodynamic, and renal indicators remains clinically relevant. Several gaps nevertheless remain. Most previous studies examined these markers individually or in partially adjusted models, and few have simultaneously estimated their independent associations together with diabetes treatment histories within a single contemporary cohort. Heart rate (HR) has received far less attention than blood pressure in DR risk assessment despite being a readily available vital sign that costs nothing to measure. Existing evidence links a faster resting HR to diabetic microvascular complications, possibly through underlying cardiac autonomic dysfunction (19), but this association has not been systematically evaluated alongside comprehensive clinical covariates in a Chinese population. Therefore, this large cross-sectional analysis examined clinical factors associated with the presence of DR, compared the cross-sectional discrimination of selected markers, and explored whether observed associations differed across HbA1c strata.

## 2 Methods

### 2.1 Data collection

This study is a cross-sectional descriptive analysis based on the China DiaChronic Study (20). A multi-stage sampling approach was employed to recruit 1,765 participants from four cities in Anhui Province, China. Data on demographic characteristics, lifestyle behaviors, and medical history were collected by trained medical personnel using standardized questionnaires. Specific variables included age, gender, smoking and alcohol consumption status, and educational level. Anthropometric measurements such as height, weight, waist circumference, blood pressure, and heart rate were also recorded. Fundus photography and other diagnostic examinations were performed. Fasting venous blood and urine samples were collected from each participant the morning after an 8-hour fast. These specimens were transported to a designated laboratory within 24 hours for biochemical analyses, including blood glucose, HbA1c, lipid profile, liver and kidney function tests. The study was conducted in full accordance with the ethical principles of the Declaration of Helsinki, as illustrated in the flowchart presented in **Figure 1**. Of the initial sample, 288 participants were excluded due to missing blood test results or inadequate fundus imaging quality; the remaining 1,477 participants constituted the complete-case analytic sample. Diabetes duration was calculated from the recorded year and month of diabetes diagnosis to the survey date. Diabetes type and treatment histories, including glucose-lowering medication use, regular insulin use, antihypertensive medication use, and lipid-lowering medication use, were extracted from the standardized questionnaire. Patient-level DR severity was defined according to the more advanced grade in the gradable eye(s) and categorized as mild, moderate, or severe non-proliferative diabetic retinopathy (NPDR) or proliferative diabetic retinopathy (PDR). Masked grading, inter-rater reliability, and OCT/OCTA-based staging were not available for the present analysis.

**Figure 1:**
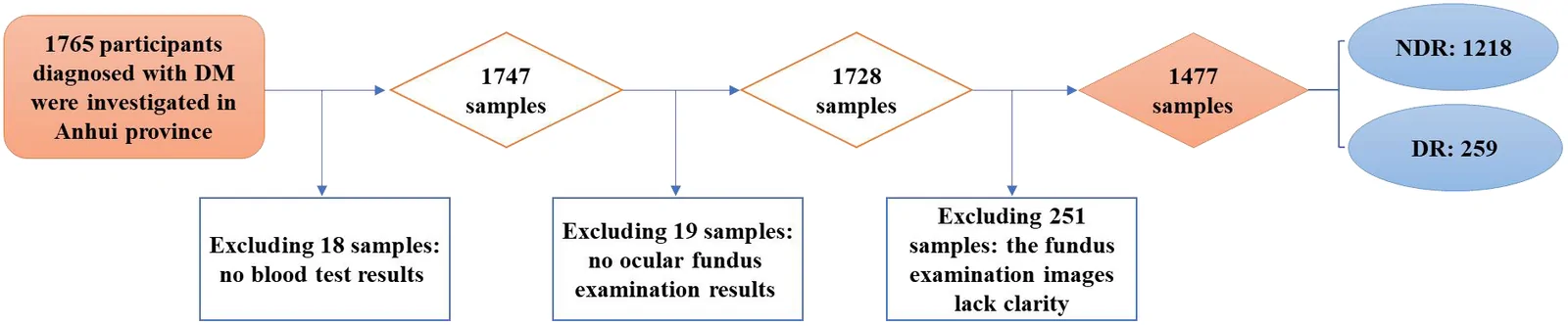
Flowchart of participants in the study.

### 2.2 Inclusion and exclusion criteria

All study participants were required to meet predetermined inclusion and exclusion criteria.

Inclusion criteria comprised: Age ≥18 years; Confirmed diagnosis of diabetes mellitus; Registered as a permanent resident in the surveyed area for ≥6 months. Exclusion criteria comprised: Current pregnancy or history of adverse pregnancy outcomes; Comorbid severe health conditions including organ failure or malignant tumors; Comorbid psychiatric disorders or intellectual disabilities impairing normal communication; Use of glucocorticoids or immunosuppressants within the past 3 months due to acute infections or chronic diseases.

### 2.3 Statistical methods

Data analysis was conducted using SPSS 23.0 software. Normality of quantitative variables was assessed with the Kolmogorov-Smirnov test; categorical variables (sex, smoking/alcohol status, education level) were expressed as frequencies (percentages) and analyzed using chi-square tests; normally distributed continuous variables were presented as mean ± standard deviation and compared via independent samples t-tests; non-normally distributed continuous variables were reported as median (*P*25, *P*75) and analyzed using Wilcoxon rank-sum tests. Multivariable analysis employed binary logistic regression to estimate associations with prevalent DR. Age, sex, smoking status, and BMI were retained as basic covariates; SBP, HR, HbA1c, TG, HDL-C, BUN, UACR, diabetes duration, glucose-lowering medication use, regular insulin use, antihypertensive medication use, and lipid-lowering medication use were included on the basis of clinical relevance and/or univariate association. Medication variables were coded as use versus non-use; participants without the corresponding pharmacological indication were classified as non-users. Diabetes type was summarized descriptively but was not entered into the primary model because it was recorded as unknown in a substantial proportion of participants. MAP was not entered with SBP because it is mathematically derived from SBP and DBP, and HbA1c was preferred over FPG in the primary model to limit redundancy. Multicollinearity was assessed using VIFs, with VIF >5 considered potentially important. Receiver operating characteristic (ROC) curves were generated only to describe cross-sectional discrimination of individual markers between participants with and without DR; they were not interpreted as prospective prediction. The HbA1c threshold of 6.5% was retained only for exploratory stratification as a diagnostic reference point rather than a universal treatment target. Separate multiplicative interaction terms were tested for HbA1c category with SBP, HR, BUN, and UACR. Because only 37 DR cases occurred in the HbA1c <6.5% stratum, subgroup results were interpreted cautiously. Spearman’s correlation analysis assessed relationships between HbA1c and clinical parameters, with visualizations created in GraphPad Prism 8.0.1. Exploratory subgroup and correlation analyses were not adjusted for multiple comparisons and were considered hypothesis-generating. Key formulas included: BMI = weight (kg)/height² (m²); MAP = DBP + ⅓ (SBP - DBP); UACR = urinary microalbumin/urinary creatinine. Statistical significance was defined as two-tailed *P* < 0.05 for all analyses. DR severity was summarized descriptively using the worse-eye grade; stage-specific multivariable regression was not performed because the severe NPDR and PDR subgroups were small.

## 3 Results

### 3.1 Factors associated with diabetic retinopathy

Among 1477 diabetic patients, fundus photography identified 259 cases with comorbid retinopathy. Based on retinopathy status, participants were stratified into two groups. Univariate analysis compared demographic characteristics (age, sex), lifestyle factors (smoking, alcohol consumption), education level, anthropometric measures, and laboratory parameters (blood/urine tests) between groups (**Table 1**). Significant intergroup differences (*P* < 0.05) were observed in systolic blood pressure (SBP), mean arterial pressure (MAP), heart rate (HR), fasting blood glucose (FBG), glycated hemoglobin (HbA1c), triglycerides, albumin, gamma-glutamyl transferase (GGT), aspartate aminotransferase (AST), blood urea nitrogen (BUN), and urinary albumin-creatinine ratio (UACR). Diabetes duration was longer in patients with DR than in those without DR [8.75 (4.67, 14.17) vs. 5.42 (3.19, 9.50) years; *P* < 0.001]. Among participants with a recorded specific diabetes type, type 2 diabetes accounted for 710/747 (95.0%); diabetes type was recorded as unknown in 730 participants. Regular insulin use was more frequent in the DR group (33.6% vs. 11.7%; *P* < 0.001), while other treatment histories are summarized in **Table 1**. Among the 259 patients with DR, 41 (15.8%) had mild NPDR, 185 (71.4%) moderate NPDR, 21 (8.1%) severe NPDR, and 12 (4.6%) PDR. After additional adjustment for diabetes duration and treatment histories (**Table 2**), higher SBP (OR 1.019, 95% CI 1.010-1.027; *P* < 0.001), HR (OR 1.016, 95% CI 1.005-1.028; *P* = 0.005), HbA1c (OR 1.207, 95% CI 1.111-1.310; *P* < 0.001), BUN (OR 1.102, 95% CI 1.017-1.195; *P* = 0.018), UACR (OR 1.00039, 95% CI 1.00007-1.00071; *P* = 0.016), and longer diabetes duration (OR 1.061, 95% CI 1.032-1.091; *P* < 0.001) were associated with prevalent DR. VIFs ranged from 1.07 to 1.59, providing no evidence of problematic multicollinearity.

**Table 1:** Univariate analysis of factors associated with diabetic retinopathy.

Variables	NDR (*n* = 1218)	DR (*n* = 259)	Statistic	*P* value
Ages, years	56.15 (50.00, 64.83)	56.70 (50.40, 64.50)	-1.156	0.248
Sex, *n* (%)			0.697	0.404
Male	593 (48.7)	134 (51.5)		
Female	625 (51.3)	126 (48.5)		
Current smoking, *n* (%)	349 (28.7)	81 (31.3)	0.711	0.399
Current alcohol drinking, *n* (%)	408 (33.5)	91 (35.1)	0.256	0.313
Educational level, *n* (%)			0.320	0.956
Under primary	539 (44.3)	116 (44.8)		
Primary	175 (14.4)	39 (15.1)		
Junior high	335 (27.5)	67 (25.9)		
Senior high above	169 (13.9)	37 (14.3)		
Diabetes type, n (%)			2.867	0.238
Type 2	575 (47.2)	135 (52.1)		
Type 1/other	29 (2.4)	8 (3.1)		
Unknown	614 (50.4)	116 (44.8)		
Diabetes duration, years	5.42 (3.19, 9.50)	8.75 (4.67, 14.17)	-7.722	<0.001
Glucose-lowering medication use, n (%)	1027 (84.3)	244 (94.2)	16.592	<0.001
Regular insulin use, n (%)	143 (11.7)	87 (33.6)	75.908	<0.001
Antihypertensive medication use, n (%)	>527 (43.3)	94 (36.3)	3.982	0.046
Lipid-lowering medication use, n (%)	190 (15.6)	25 (9.7)	5.604	0.018
BMI, kg/m²	25.80 (23.70, 28.00)	25.50 (23.20, 27.70)	-1.433	0.152
WC, cm	89.61 ± 9.91	89.66 ± 9.82	-0.076	0.939
SBP, mmHg	142.00 (129.67, 155.08)	148.43 ± 18.91	-4.061	<0.001
DBP, mmHg	82.00 (74.59, 89.67)	82.42 ± 10.17	-0.123	0.902
MAP, mmHg	102.22 (94.41, 110.33)	104.42 ± 11.45	-2.177	0.029
HR, bpm	77.67 (70.67, 86.67)	81.33 (73.67, 90.33)	-4.080	<0.001
Laboratory results				
FPG, mmol/L	8.34 (6.95, 10.49)	10.23 (7.98, 13.26)	-7.553	<0.001
HbA1c, %	7.00 (6.20, 8.30)	8.30 (7.00, 9.60)	-8.704	<0.001
TC, mmol/L	5.12 (4.41, 5.80)	5.12 (4.39, 5.87)	-0.418	0.676
TG, mmol/L	1.75 (1.24, 2.53)	1.60 (1.10, 2.51)	-2.038	0.042
LDL-C, mmol/L	3.02 (2.40, 3.68)	3.03 (2.46, 3.58)	-0.022	0.983
HDL-C, mmol/L	1.36 (1.14, 1.62)	1.40 (1.15, 1.72)	-1.588	0.112
Alb, g/L	51.67 ± 3.08	51.50 (49.00, 53.20)	-2.425	0.015
GGT, IU/L	28.00 (18.00, 47.00)	24.00 (17.00, 44.00)	-2.714	0.007
AST, IU/L	20.00 (17.00, 27.00)	19.00 (16.00, 25.00)	-2.514	0.012
ALT, IU/L	21.00 (15.00, 31.00)	20.00 (15.00,27.00)	-1.681	0.093
AST/ALT	0.95(0.78,1.21)	0.94(0.79, 1.21)	-0.283	0.777
Scr, mmol/L	67.00 (57.00, 78.00)	70.00 (56.00, 80.00)	-0.845	0.398
BUN, mmol/L	5.50 (4.60, 6.60)	6.60 (5.10, 7.30)	-4.991	<0.001
UA, mmol/L	297.00 (249.00, 360.00)	293.00 (250.00, 359.00)	-0.200	0.841
UACR, mg/g	18.55 (7.22, 49.84)	48.34 (13.21, 154.47)	-7.831	<0.001

Data are presented as means ± standard deviation, means (*P*25, *P*75) or numbers (%). NDR, non-diabetic retinopathy; DR, diabetic retinopathy; BMI, body mass index; WC, waist circumference; SBP, systolic blood pressure; DBP, diastolic blood pressure; MAP, mean arterial pressure; HR, heart rate; FPG, fasting plasma glucose; HbA1c, glycosylated hemoglobin; TC, total cholesterol; TG, triglycerides; LDL-C, low density lipoprotein cholesterol; HDL-C, high density lipoprotein cholesterol; Alb, albumin; GGT, γ-glutamyl transpeptidase; AST, aspartate aminotransferase; ALT, alanine aminotransferase; Scr, serum creatinine; BUN, blood urea nitrogen; UA, uric acid; UACR, urinary albumin-creatinine ratio. Diabetes type was recorded as type 1, type 2, other specific type, or unknown; “Type 1/other” combines type 1 (*n* = 34) and other specific types (*n* = 3). Medication-use variables indicate reported use at the time of assessment.

**Table 2:** Multivariable logistic regression of factors associated with prevalent diabetic retinopathy.

Variables	*β*	SE	Wald χ²	*P* value	OR	95% CI
Age, years	0.003	0.009	0.085	0.770	1.003	0.986-1.020
Sex (female vs male)	0.033	0.189	0.030	0.864	1.033	0.713-1.497
Current smoking	0.164	0.203	0.653	0.419	1.178	0.792-1.752
BMI, kg/m²	-0.012	0.023	0.297	0.586	0.988	0.945-1.033
SBP, mmHg	0.018	0.004	18.456	<0.001	1.019	1.010-1.027
HR, bpm	0.016	0.006	7.729	0.005	1.016	1.005-1.028
HbA1c, %	0.188	0.042	19.939	<0.001	1.207	1.111-1.310
TG, mmol/L	-0.018	0.034	0.269	0.604	0.983	0.919-1.050
HDL-C, mmol/L	0.195	0.185	1.110	0.292	1.216	0.845-1.748
BUN, mmol/L	0.097	0.041	5.564	0.018	1.102	1.017-1.195
UACR, mg/g	0.00039	0.00016	5.843	0.016	1.00039	1.00007-1.00071
Diabetes duration, years	0.059	0.014	17.737	<0.001	1.061	1.032-1.091
Glucose-lowering medication use	0.769	0.306	6.332	0.012	2.158	1.185-3.929
Regular insulin use	0.765	0.185	17.134	<0.001	2.148	1.496-3.085
Antihypertensive medication use	-0.568	0.175	10.500	0.001	0.567	0.402-0.799
Lipid-lowering medication use	-0.579	0.253	5.233	0.022	0.561	0.341-0.920

BMI, body mass index; SBP, systolic blood pressure; HR, heart rate; HbA1c, glycosylated hemoglobin; TG, triglycerides; HDL-C, high density lipoprotein cholesterol; BUN, blood urea nitrogen; UACR, urinary albumin-creatinine ratio; OR, odds ratio; CI, confidence interval. Diabetes duration and medication-history variables were included as adjustment covariates. Medication coefficients should not be interpreted as causal treatment effects.

### 3.2 Cross-sectional discrimination of selected clinical markers

ROC analysis was used descriptively to compare cross-sectional discrimination between participants with and without prevalent DR (**Figure 2**). HbA1c had the largest AUC (0.672, 95% CI 0.637-0.707), followed by UACR (0.655, 95% CI 0.617-0.692), BUN, HR, SBP, and MAP (**Table 3**). These AUCs indicate only modest discrimination and do not demonstrate prospective predictive performance.

**Figure 2:**
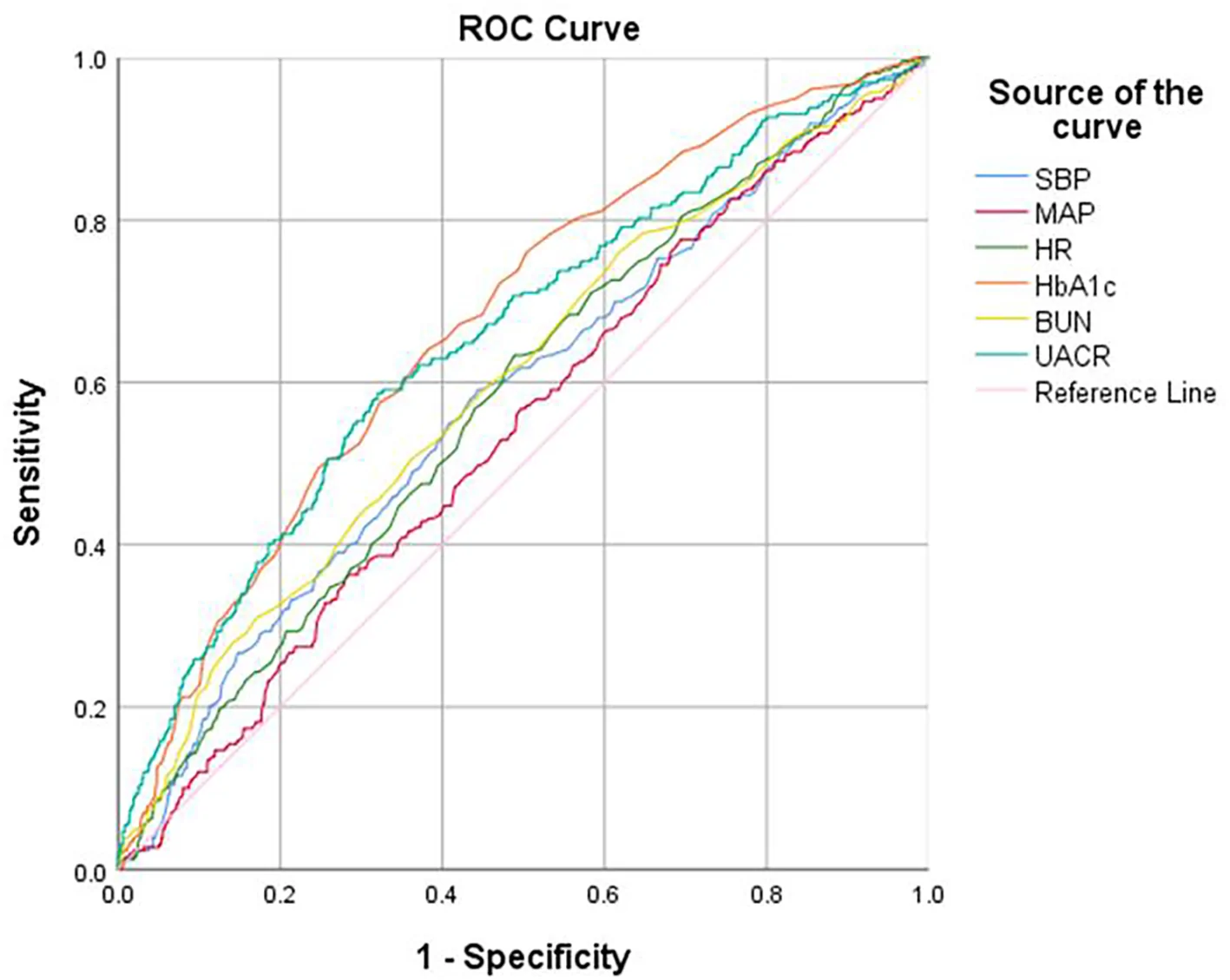
ROC analyses for cross-sectional discrimination of diabetic retinopathy.

**Table 3:** Cross-sectional ROC discrimination of selected clinical markers.

Variables	AUC (95% CI)	Cut-off	Sensitivity	Specificity
SBP, mmHg	0.580 (0.542-0.619)	143.835	0.591	0.557
MAP, mmHg	0.543 (0.505-0.581)	96.275	0.776	0.306
HR, bpm	0.581 (0.543-0.618	78.165	0.633	0.511
HbA1c, %	0.672 (0.637-0.707)	7.550	0.641	0.617
BUN, mmol/L	0.599 (0.560-0.637)	5.950	0.506	0.638
UACR, mg/g	0.655 (0.617-0.692)	34.973	0.587	0.680

SBP, systolic blood pressure; MAP, mean arterial pressure; HR, heart rate; HbA1c, glycosylated hemoglobin; BUN, blood urea nitrogen; UACR, urinary albumin-creatinine ratio; AUC, area under the ROC curve.

### 3.3 Exploratory analyses stratified by HbA1c

For exploratory analysis, participants were stratified by HbA1c <6.5% and ≥6.5%; the 6.5% value was treated as a diagnostic reference point rather than a universal glycemic-control target. DR prevalence was 8.3% (37/446) in the HbA1c <6.5% stratum and 21.5% (222/1,031) in the HbA1c ≥6.5% stratum (*P* < 0.05). Univariate analysis (**Table 4**) indicated that BMI, FBG, and low-density lipoprotein cholesterol (LDL-C) were significant factors (*P* < 0.05) in the HbA1c <6.5% stratum, whereas SBP, MAP, HR, FBG, triglycerides (TG), high-density lipoprotein cholesterol (HDL-C), albumin, GGT, AST, alanine aminotransferase (ALT), BUN and UACR showed significance (*P* < 0.05) in the HbA1c ≥6.5% stratum. Because only 37 DR cases occurred in the HbA1c <6.5% stratum, subgroup estimates were considered underpowered. Formal interaction tests showed no statistically significant evidence that HbA1c category modified the associations of SBP (*P* for interaction = 0.090), HR (*P* for interaction = 0.231), BUN (*P* for interaction = 0.170), or UACR (*P* for interaction = 0.309) with prevalent DR (**Table 5**).

**Table 4:** Univariate analysis of diabetic retinopathy in patients stratified by HbA1c levels (<6.5% vs. ≥6.5%).

Variables	HbA1c <6.5%				HbA1c ≥6.5%			
	NDR (*n* = 409)	DR (*n* = 37)	Statistic	*P* value	NDR (*n* = 809)	DR (*n* = 222)	Statistic	*P* value
Ages, years	55.60 (48.80, 64.65)	58.34 ± 8.50	-1.352	0.176	56.40 (50.10,64.95)	56.70 (50.30, 64.35)	-0.390	0.697
Sex, *n* (%)			0.898	0.343			<0.001	0.985
Male	177 (43.3)	19 (51.4)			416 (51.4)	114 (51.4)		
Female	232 (56.7)	18 (48.6)			393 (48.6)	108 (48.6)		
Current smoking, *n* (%)	98 (24.0)	13 (35.1)	2.266	0.132	251 (31.0)	68 (30.6)	0.013	0.910
Current alcohol drinking, *n* (%)	135 (33.0)	14 (37.8)	0.356	0.551	273 (33.7)	77 (34.7)	0.069	0.793
Educational level, *n* (%)			0.282	0.963			1.328	0.722
Under primary	182 (44.5)	17 (45.9)			357 (44.1)	99 (44.6)		
Primary	57 (13.9)	4 (10.8)			118 (14.6)	35 (15.8)		
Junior high	96 (23.5)	9 (24.3)			239 (29.5)	58 (26.1)		
Senior high above	74 (18.1)	7 (18.9)			95 (11.7)	30 (13.5)		
BMI, kg/m²	25.50 (23.40, 27.50)	24.30 (21.65, 26.30)	-2.251	0.024	25.90 (23.80, 28.20)	25.89 ± 3.66	-1.076	0.282
WC, cm	87.50 (80.90, 94.05)	86.61 ± 8.97	-0.908	0.364	90.38 ± 9.69	90.17 ± 9.88	0.868	0.352
SBP, mmHg	140.67 (128.33, 154.84)	142.31 ± 15.76	-0.414	0.679	142.33 (131.00, 155.67)	149.45 ± 19.22	-3.961	<0.001
DBP, mmHg	82.43 ± 11.48	79.19 ± 9.66	1.542	0.215	82.51 ± 10.79	82.96 ± 10.18	1.661	0.198
MAP, mmHg	102.48 ± 12.55	100.23 ± 10.89	0.92	0.388	102.33 (94.89, 110.33)	105.12 ± 11.42	-2.516	0.012
HR, bpm	76.33 (69.67, 84.00)	79.33 (73.67, 93.33)	-1.989	0.047	79.00 (71.17, 87.67)	81.67 (73.59, 89.75)	-2.907	0.004
Laboratory results								
FPG, mmol/L	6.75 (5.96, 7.63)	7.57 ± 1.51	-2.448	0.014	9.58 (8.04, 11.63)	10.94 (8.84, 13.97)	-4.813	<0.001
TC, mmol/L	5.16 (4.37, 5.81)	4.82 (4.27, 5.21)	-1.605	0.109	5.09 (4.43, 5.79)	5.25 (4.41, 5.90)	-0.998	0.318
TG, mmol/L	1.58 (1.13, 2.29)	1.43 (1.12, 2.10)	-0.366	0.714	1.84 (1.28, 2.67)	1.64 (1.09, 2.55)	-2.705	0.007
LDL-C, mmol/L	3.06 ± 0.97	2.78 ± 0.73	6.516	0.011	3.01 (2.43, 3.68)	3.07 (2.46, 3.62)	-0.672	0.501
HDL-C, mmol/L	1.43 (1.19, 1.70)	1.44 (1.34, 1.61)	-0.512	0.609	1.33 (1.11, 1.59)	1.40 (1.17, 1.74)	-2.569	0.010
Alb, g/L	51.92 ± 3.03	51.85 ± 2.76	0.223	0.637	51.54 ± 3.10	51.35 (49.00, 53.03)	-2.253	0.024
GGT, IU/L	24.00 (17.00, 40.00)	25.00 (13.50, 44.00)	-0.815	0.415	29.00 (19.50, 51.00)	24.00 (17.00, 44.00)	-3.384	0.001
AST, IU/L	21.00 (17.00, 26.00)	21.00 (16.50, 28.00)	-0.345	0.730	20.00 (16.00, 27.00)	19.00 (15.00, 24.00)	-2.543	0.011
ALT,IU/L	20.00 (14.00, 30.00)	20.00 (14.00, 27.50)	-0.693	0.488	22.00 (16.00, 32.00)	20.00 (15.75, 27.00)	-2.071	0.038
AST/ALT	1.04 (0.83, 1.29)	1.27 ± 0.55	-1.728	0.084	0.92 (0.76, 1.15)	0.92 (0.79, 1.18)	-0.702	0.483
Scr, mmol/L	68.00 (58.00, 81.00)	70.92 ± 20.50	-0.085	0.933	66.00 (57.00, 78.00)	70.00 (56.00, 80.25)	-1.232	0.218
BUN, mmol/L	5.30 (4.50, 6.40)	5.58 ± 1.62	-0.306	0.759	5.50 (4.70, 6.60)	6.10 (5.20, 7.40)	-5.035	<0.001
UA, mmol/L	313.00 (260.50, 373.00)	326.65 ± 82.68	-0.463	0.643	290.00 (243.00, 351.00)	289.50 (246.00, 357.25)	-0.382	0.702
UACR, mg/g	12.14 (5.37, 30.89)	16.80 (7.27, 39.55)	-1.165	0.244	22.77 (8.46, 61.31)	52.09 (17.21, 198.06)	-6.943	<0.001

Data are presented as means ± standard deviation, means (P25, P75) or numbers (%). NDR, non-diabetic retinopathy; DR, diabetic retinopathy; BMI, body mass index; WC, waist circumference; SBP, systolic blood pressure; DBP, diastolic blood pressure; MAP, mean arterial pressure; HR, heart rate; FPG, fasting plasma glucose; HbA1c, glycosylated hemoglobin; TC, total cholesterol; TG, triglycerides; LDL-C, low density lipoprotein cholesterol; HDL-C, high density lipoprotein cholesterol; Alb, albumin; GGT, γ-glutamyl transpeptidase; AST, aspartate aminotransferase; ALT, alanine aminotransferase; Scr, serum creatinine; BUN, blood urea nitrogen; UA, uric acid; UACR, urinary albumin-creatinine ratio.

**Table 5:** Exploratory interaction tests by HbA1c category.

Exploratory interaction term	*P* for interaction
SBP × HbA1c category	0.090
HR × HbA1c category	0.231
BUN × HbA1c category	0.170
UACR × HbA1c category	0.309

BUN, blood urea nitrogen; HbA1c, glycosylated hemoglobin; HR, heart rate; SBP, systolic blood pressure; UACR, urinary albumin-creatinine ratio. Interaction P values were obtained from separate multiplicative interaction terms between HbA1c category (<6.5% vs ≥6.5%) and each selected clinical variable. These analyses were exploratory.

### 3.4 Correlation of HbA1c with selected clinical variables in participants with HbA1c ≥6.5%

Within the HbA1c ≥6.5% stratum, we examined concurrent correlations between HbA1c and selected clinical variables. The correlation analysis (**Figure 3**) revealed that FBG, HR, BUN and UACR were positively correlated with HbA1c levels (*r* = 0.094-0.641, *P* < 0.05). Among these, besides FBG, the UACR showed the strongest correlation (*r* = 0.216, *P* < 0.001), followed by HR and BUN. These correlations are cross-sectional and do not establish temporal or causal relationships.

**Figure 3:**
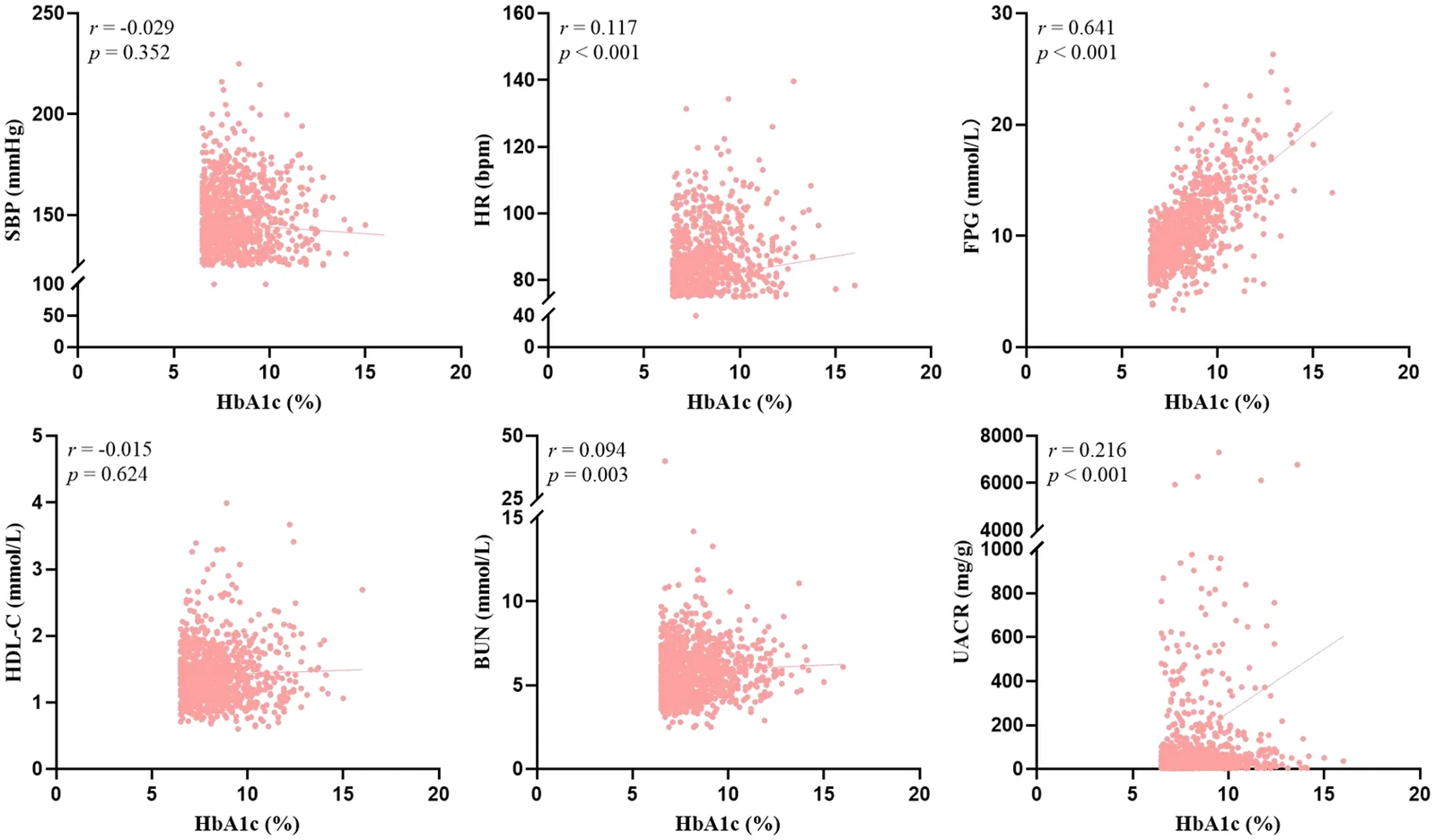
Correlation analysis between clinical variables and hba1c levels in patients with HbA1c ≥6.5%.

## 4 Discussion

Despite increasingly sophisticated prevention and management systems for diabetes, DR remains an unignorable complication for patients with diabetes. It significantly impacts patients’ quality of life and poses a substantial burden on healthcare systems. Identifying clinical factors associated with prevalent DR is relevant to comprehensive diabetes care and ophthalmic assessment. This cross-sectional descriptive study analyzed data from 1477 patients with diabetes (including 259 with DR). It examined factors associated with prevalent DR while additionally accounting for diabetes duration and major medication histories, described the cross-sectional discrimination of selected markers, and performed exploratory HbA1c-stratified analyses with formal interaction testing. Although the individual associations reported here largely corroborate established risk factors, the study adds to the literature in several specific ways. It simultaneously quantifies the mutually adjusted associations of glycemic, hemodynamic, and renal indicators together with treatment histories in a large contemporary Chinese cohort; it evaluates HR, an understudied and readily available vital sign, alongside comprehensive covariates; and it formally tests whether these associations are modified by HbA1c category.

In the expanded multivariable model, higher SBP, HR, HbA1c, BUN, UACR, and longer diabetes duration were associated with prevalent DR after adjustment for major treatment histories. As the gold standard for assessing glycemic control, elevated HbA1c promotes the accumulation of AGEs (21), induces oxidative stress (22), and ultimately leads to dysfunction of the retinal neurovascular unit (23). Consistent with previous studies (24), the present findings are consistent with an association between higher HbA1c and prevalent DR, highlighting the central role of sustained hyperglycemia in retinal microvascular damage. Longer diabetes duration was also independently associated with prevalent DR, supporting the importance of cumulative metabolic exposure in retinal microvascular injury. Treatment-history variables were incorporated primarily to reduce confounding; their regression coefficients should not be interpreted as causal treatment effects because confounding by indication and disease severity remains possible. Higher SBP may reflect greater hemodynamic burden. In the original model, SBP and MAP were entered together and showed opposite coefficient directions. Because MAP is mathematically derived from SBP and DBP, the revised primary model retained SBP and excluded MAP; all VIFs were below 2. The remaining positive association of SBP with DR is consistent with previous evidence linking blood-pressure burden to diabetic microvascular disease (25–27) and underscore the necessity for comprehensive blood pressure management in diabetic patients. Elevated BUN and UACR reflect impaired microvascular filtration function in the kidneys (28). Given the anatomical and physiological similarities between the microvasculature of the retina and kidneys, concurrent damage to both systems underscores the systemic nature of diabetic microangiopathy. While UACR, a sensitive marker of early renal injury, has previously been linked to DR (29), the present multivariable analysis showed an independent cross-sectional association with DR. HR was also associated with prevalent DR. However, heart rate is influenced by autonomic neuropathy, cardiovascular disease, medications, physical activity, anxiety, thyroid status, and measurement conditions; therefore, it should not be interpreted as a novel causal determinant on the basis of the present cross-sectional data (30). The association between higher HR and prevalent DR, although modest in magnitude, is the most potentially novel finding in this study. A faster resting HR has previously been associated with a higher risk of microvascular complications, including retinopathy, in a large international cohort of patients with type 2 diabetes (19), and cardiac autonomic neuropathy (CAN), a condition characterized in part by resting tachycardia and reduced heart-rate variability, has been consistently linked to DR (31). These findings suggest that resting HR may partly serve as a readily available surrogate for underlying cardiac autonomic dysfunction and sympathetic overactivity, both of which cluster with retinal and renal microvascular damage in diabetes. This interpretation requires caution, however, because HR is influenced by autonomic neuropathy, cardiovascular disease, medications, physical activity, anxiety, thyroid status, and measurement conditions, and residual confounding by these factors cannot be excluded in a cross-sectional design (30). HR should therefore not be regarded as a novel causal determinant of DR. It is more appropriately viewed as a simple, cost-free clinical sign that may help identify patients who warrant closer attention to glycemic, hemodynamic, and renal risk factors and to the completeness of recommended retinal screening.

ROC analysis showed that HbA1c had the largest AUC, followed by UACR; however, both markers demonstrated only modest cross-sectional discrimination. Because these analyses used prevalent DR at a single time point and no prediction model was internally or externally validated, the AUCs should not be interpreted as evidence of future predictive performance. The association of UACR with DR is compatible with shared retinal and renal microvascular injury (32), but it does not establish UACR as an early retinal biomarker or a stand-alone screening test.

HbA1c levels may change with pharmacologic and lifestyle interventions (33, 34); however, 6.5% is a conventional diagnostic reference point rather than a universal glycemic treatment target. Accordingly, the <6.5% and ≥6.5% categories were retained only for exploratory stratification and are not described as “target-achieved” and “target-not-achieved” groups. More variables reached statistical significance within the HbA1c ≥6.5% stratum, but the HbA1c <6.5% stratum contained only 37 DR cases and had limited statistical power. Moreover, formal interaction tests for SBP, HR, BUN, and UACR were not statistically significant. Therefore, the subgroup findings do not demonstrate that HbA1c category modifies these associations or that lower HbA1c abolishes the contribution of other clinical factors (35).

Within the HbA1c ≥6.5% stratum, correlation analysis revealed positive associations between HR, BUN, UACR, and HbA1c. Among these, UACR demonstrated the strongest correlation after FBG. This finding further corroborates the existence of shared pathological pathways underlying microvascular damage in the retina and kidneys (e.g., AGEs accumulation, oxidative stress). UACR is an established marker of renal injury (36, 37), but its cross-sectional association with DR does not establish it as an early retinal biomarker. The present study also did not construct or validate a combined HbA1c-UACR prediction model; therefore, no claim is made that combining these measures improves future DR prediction. Collectively, this research underscores the close connections among DR, cardiovascular and renal function, and metabolic dysregulation. Clinically, these findings support attention to coexisting glycemic, cardiovascular, renal, and ophthalmic abnormalities within comprehensive diabetes care. The parsimonious set of indicators identified here, namely diabetes duration, HbA1c, SBP, HR, BUN, and UACR, can all be obtained from routine clinical encounters without advanced imaging, and HR in particular is available at essentially no cost. Their simultaneous association with prevalent DR therefore supports a multidimensional, low-cost approach to flagging patients who may benefit from closer attention to glycemic, hemodynamic, and renal risk factors and to the completeness of recommended retinal screening, while screening intervals and treatment decisions should continue to follow established clinical guidance.

This study has several limitations. First, the cross-sectional design identifies associations with prevalent DR but cannot establish temporality, causality, future prediction, or DR progression. Second, although diabetes duration and major medication histories were incorporated, diabetes type was recorded as unknown in 730 participants, and genetic predisposition, diet, physical activity, and detailed renal disease history remained incompletely characterized; residual confounding therefore remains possible. In particular, resting HR is susceptible to residual confounding by autonomic neuropathy, cardiovascular disease, medication use, physical activity, anxiety, and thyroid status, which were not comprehensively captured; the HR finding should therefore be interpreted with particular caution. Third, fundus grades enabled descriptive DR severity classification, but the severe NPDR and PDR subgroups were small (*n* = 21 and *n* = 12, respectively), limiting stable stage-specific multivariable analyses. Masked grading, inter-rater reliability measures, and OCT/OCTA-based staging were not available. Fourth, exploratory subgroup and correlation analyses involved multiple comparisons without multiplicity correction, and the HbA1c <6.5% stratum contained only 37 DR cases. Fifth, participants were recruited from four cities in Anhui Province, which may limit generalizability. Prospective studies with more complete diabetes-type ascertainment, detailed treatment exposure, standardized multimodal DR grading, and longitudinal outcomes are needed to establish temporal relationships and develop validated risk-prediction models.

Based on questionnaire data from a large sample of 1,477 patients, this study examined factors associated with prevalent DR, described cross-sectional discrimination, and conducted exploratory analyses by HbA1c category. The formal interaction analyses did not demonstrate effect modification by HbA1c category. Overall, the study provides contemporary real-world evidence that the mutually adjusted associations of established risk factors remain robust in a regional Chinese population. It also draws attention to resting HR an understudied and readily available marker that may complement existing indicators within a low-cost risk-assessment strategy. Although the study lacks the causal inference capabilities of a prospective cohort design, the findings should reinforce rather than replace established DR screening recommendations. Patients with longer diabetes duration, higher HbA1c, elevated blood pressure, elevated HR, or evidence of renal microvascular injury such as increased UACR may warrant particular attention to whether recommended retinal examinations have been completed, while screening intervals and treatment decisions should continue to follow established clinical guidance and individual patient characteristics.

## 5 Conclusions

In this cross-sectional study of 1,477 patients with diabetes, higher SBP, HR, HbA1c, BUN, UACR, and longer diabetes duration were associated with prevalent DR after adjustment for demographic, metabolic, and treatment-related covariates. Among these, the association with HR, a cost-free and readily available vital sign, was the most potentially novel and may reflect cardiac autonomic dysfunction rather than a direct causal effect. HbA1c and UACR showed modest cross-sectional discrimination, while the combined pattern of glycemic, hemodynamic, renal, and diabetes-duration associations supports a multidimensional approach to identifying patients who may warrant closer attention to guideline-recommended retinal assessment. These findings strengthen real-world evidence for an integrated cardiometabolic, renal, and ophthalmic assessment that complements rather than replaces established screening integrated DR risk assessment and should be validated prospectively (**Box 1**).

Text box 1. Contributions to the Literature

- Examines routinely available glycemic, hemodynamic, and renal markers, together with diabetes treatment histories, associated with prevalent DR in a large cross-sectional sample.
- Highlights HR, an understudied and cost-free vital sign, as the most potentially novel association, while cautioning against a causal interpretation.
- Clarifies that HbA1c and UACR have only modest cross-sectional discriminatory ability and are not validated prospective predictors.
- Supports integrated cardiometabolic, renal, and ophthalmic diabetes and ophthalmic assessment without proposing new causal relationships or screening thresholds.

## Data Availability

The raw data supporting the conclusions of this article will be made available by the authors, without undue reservation.
